# Non-traumatic chief complaints in older patients in European emergency departments and associated outcomes during the Covid-19 pandemic – a secondary analysis of the cross sectional EGERS study

**DOI:** 10.1186/s12877-026-08158-3

**Published:** 2026-09-05

**Authors:** Dorothee Riedlinger, Mehmet A. Karamercan, Zerrin D. Dündar, Kelly Janssens, Visnja N. Adam, Lars P. Bjørnsen, Andrea Fabbri, Said Laribi, Martin Möckel, Effie Polyzogopoulou, Sandor Somodi, Mirac Koc, Tamer Colak, Anna Slagman, the EGERS study investigators, Anne-Sophie Bard, Damien Viglono, Emmanuel Montassier, Marie Drogrey, Nicolas Marjanovic, Tahar Chouihed, Carlos El Khoury, Mehmet Ergin, Hüseyin Avni Demir, Fulya Kose, Sebastian Wolfrum, Domagoj Schunk, Arina Kruis, Prodeep Mukherjee, Denys Shchetkovskyy, Aine Mitchell, Rosa McNamara, Beniamino Susi, Szabolcs Gaál, Szilvia Németh-Simon, István Karóczkai, Andrea Válint, Ivan Juríc, Marina Pavletić, Maria Gamvroudi

**Affiliations:** 1 Division of Emergency Medicine, Charité – Universitätsmedizin Berlin, corporate member of Freie Universität Berlin and Humboldt-Universität Zu Berlin, Campus Virchow-Klinikum and Campus Charité Mitte, Berlin, Germany; 2 Department of Emergency Medicine, Faculty of Medicine, Gazi University, Ankara, Turkey; 3 Department of Emergency Medicine, Faculty of Medicine, Necmettin Erbakan University, Konya, Turkey; 4 Department of Emergency Medicine, St. Michaels Hospital, Dublin, Ireland; 5 Department of Anesthesiology, Resuscitation and Intensive Care, Clinical Hospital Sveti Duh, Zagreb, Croatia; 6 Department of Circulation and Medical Imaging, Norwegian University of Science and Technology (NTNU), Trondheim, Norway; 7 Ipartimento Emergenza, Azienda USL Della Romagna, Forli, Italy; 8 Emergency Medicine Department, Tours University Hospital, Tours, France; 9 Emergency Medicine Department National and Kapodistrian University of Athens, Attikon University Hospital, Athens, Greece; 10 Faculty of Medicine, University of Debrecen, Debrecen, Hungary; 11 Department of Emergency Medicine, Faculty of Medicine, University of Kastamonu, Kastamonu, Turkey; 12 Department of Emergency Medicine, Faculty of Medicine, Bolu Abant Izzet Baysal University, Bolu, Turkey

**Keywords:** Emergency department, Chief complaint, Geriatric patient, Geriatric emergency medicine, Mortality

## Abstract

**Background:**

The scope of medical conditions addressed in Emergency Departments (ED) increases with growing numbers of older patients among the ED patient population. More information on their chief complaints and related treatment outcomes is essential to ensure high-quality ED care. The objective of this study was to describe the pattern of chief complaints of older ED patients and evaluate the associations between these complaints and outcomes such as mortality and hospital admission.

**Methods:**

This secondary analysis is based on the observational ‘Epidemiology of Geriatric patients in Europe’ (EGERS) study. This prospective, routine data cross-sectional study was conducted in 36 EDs across 9 European countries. Data collection from routine clinical records of all patients aged 65 and older over a 7-day period from October 19 to November 30, 2020 during the Covid-19 pandemic. Patients with one documented non-traumatic chief complaint were analyzed. Descriptive statistics, logistic regression, and general linear mixed models were used to assess factors associated with hospital admission and in-hospital mortality. Odds ratios (OR) and 95% confidence intervals (CI) were reported.

**Results:**

The study population consisted of 2,794 patients with one specified chief complaint. The mean age was 77.4 (± 8.2) years. The most frequent chief complaints were shortness of breath, abdominal pain, chest pain, weakness and fatigue, and extremity pain and numbness, representing 59% of the cohort. Highest age (≥ 85), geriatric risks (i.e. presence of dementia/Alzheimer’s disease (AD), home help service or previous falls), polypharmacy, and shortness of breath increased the odds of being admitted to hospital (OR 1.4 [1.2–1.9]; OR 1.6 [1.3–1.9]; OR 1.3 [1.1–1.6]; OR 3.0 [2.4–3.8]), while female sex was negatively associated with admission (OR 0.7 [0.6–0.8]). Shortness of breath was positively associated with in-hospital mortality (OR 2.2 [1.4–4.3]).

**Conclusion:**

Older patients frequently require hospital admission. As chief complaints do not reflect underlying condition or severity, structured evaluation, staff training and geriatric specific care pathways may improve care and reduce adverse clinical outcomes.

**Supplementary Information:**

The online version contains supplementary material available at https://doi.org/10.1186/s12877-026-08158-3.

## Background

The proportion of older people and the average life expectancy in the overall population are increasing in Europe and worldwide. Accordingly, the number of emergency department (ED) visits in patients of advanced age is rising [1, 2]. The treatment of older patients adds specific challenges to ED care. Their less pronounced or non-specific symptom presentation due to age-related physiological changes increases the risk of under-triage and misdiagnosis [3–7]. Older patients have specific needs and their care requires a broad scope of medical knowledge, including but not limited to age-related and chronic health conditions; associations of chief complaints with medical conditions; risks arising with ED procedures; and geriatric risk indicators (care dependency, recurrent falls, or polypharmacy) and their influence on treatment and outcomes [8–13].

The chief complaint mandates the care protocol for patients in the ED. The scientific rationale for care protocols mostly stems from research that excludes older or multi-morbid individuals. Increasing knowledge on age-specific ED presentations and raising awareness of the need to integrate geriatric aspects in the ED care of older patients is crucial to ensure high quality medical care for this growing population. Previous studies highlighted non-specific complaints as a significant presentation in older adults associated with adverse outcomes [4, 14, 15] and showed that triage in ED tend to underestimate the acuity of specific symptoms in older patients, e.g.in abdominal pain [16].

Little is known about general patterns of chief complaints and associated diagnoses or outcome parameters, such as hospital admission or mortality rates, in unselected older ED patients throughout Europe. This study reports the frequencies of main complaints in ED patients of higher age and identifies risk factors associated with admission and in-hospital mortality.

## Methods

This secondary analysis of the cross-sectional ‘Epidemiology of Geriatric Patients Presenting to Emergency Departments in Europe’ (EGERS) study focuses on presenting chief complaints in the ED of patients 65 years and older. Data collection from routine clinical records took place prospectively for seven consecutive days between October 19th and November 30th 2020. Corresponding periods were used in each of the nine countries of the 36 participating EDs. Only the first visit of any individual was included. The study design and variable set were published before [17]. Ethic committees’ approval was obtained at all study sites and study procedures followed the principals of the Declaration of Helsinki. The trial number at the US National Library of Medicine Clinical Trials is NCT04680299.

### Study population

**Fig. 1:**
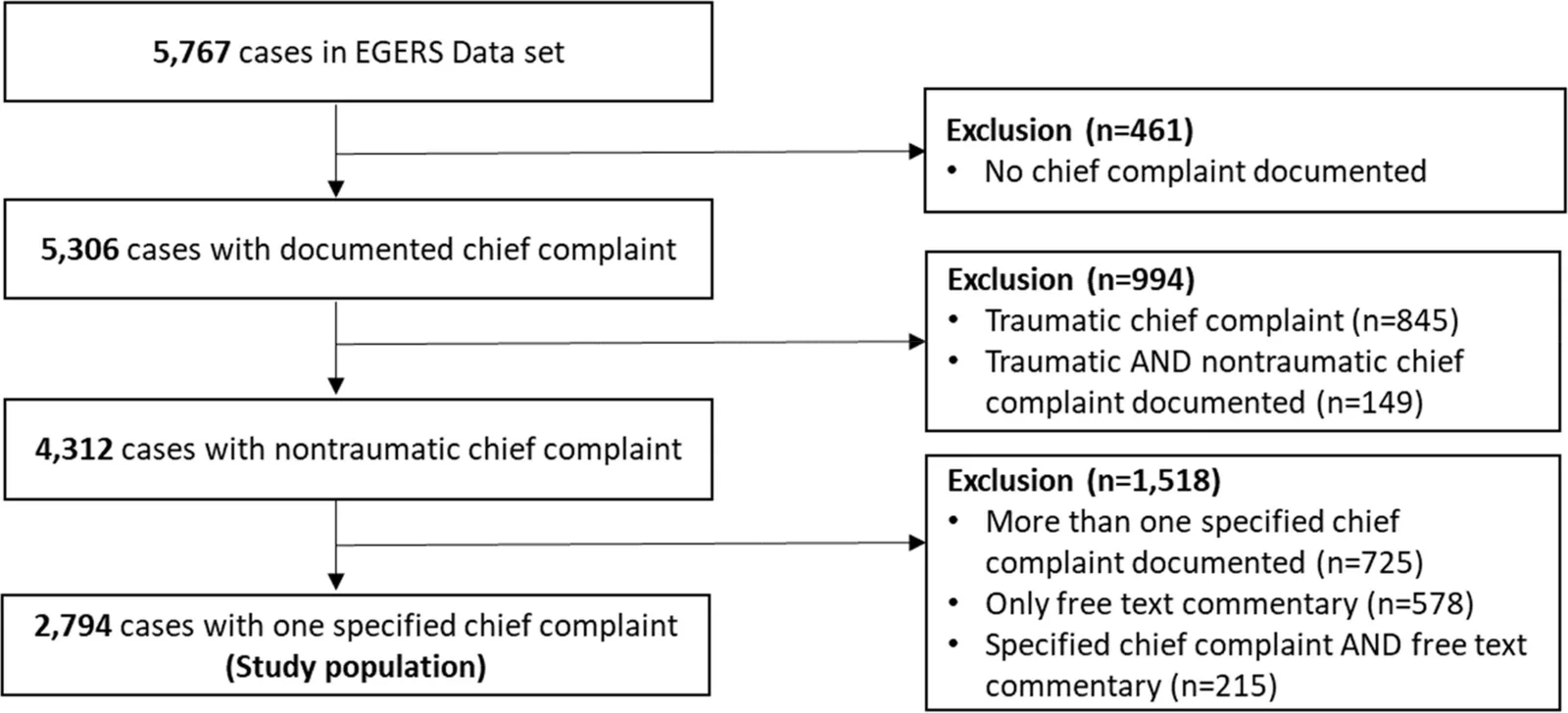
Flow chart of the inclusion and exclusion criteria

This analysis included all patients age 65 or older presenting with a single non-traumatic chief complaint of the EGERS cohort. Figure 1 displays the inclusion and exclusion criteria. The study period corresponded in many participating countries to an intense activity of the Covid-19 pandemic with potential effects on the distribution and outcomes of presenting complaints and the health-care utilization as reported in several studies [18, 19].

### Variables and measures

Variables included in the analysis were gender, age, day and time of admission to ED, chief complaints (based on a predefined selection), comorbidities, chronic medication, home help-service utilization, previous falls, information on admission to the hospital or to an intensive care unit (ICU), length of stay (LOS) in ED and hospital, and in-hospital mortality. Not all participating EDs contributed data on ED observation unit length of stay, so this variable was excluded from analysis.

Age was divided into three age groups: 65–74 years, 75–84 years and ≥ 85 years. Patients were defined as multi-morbid if more than two chronic diseases were documented following the definition of van den Bussche [20]. Polypharmacy was assumed if five or more regular medications were documented. The predefined set of chief complaints did not include urologic or stroke-like/neurologic symptoms. Consequently, patients presenting to the ED for these reasons were not captured in this analysis.We defined two risk categories: cardiovascular risk (having coronary artery disease (CAD), prior coronary revascularization, diabetes mellitus, hypertension, chronic dyslipidemia, and/or smoking), geriatric risk indicators (presence of dementia/Alzheimer’s disease (AD), home help service or previous falls). Frailty was not included in the clinical report form of the EGERS dataset, the geriatric risk indicators were used as a pragmatic approximation but do not include distinct constructs of frailty. Each of these risks was considered elevated if at least one of the defined variables applied to a patient.

### Study endpoints

The primary objective was to describe the distribution and associated mortality rates of non-traumatic chief complaints at hospital admission. The secondary objective was to evaluate the association of chief complaints and risk factors with in-hospital mortality for all admitted patients and the association of chief complaint and geriatric risk profiles with hospital admission.

### Statistical analysis

For descriptive statistical analysis, frequencies and proportions as well as 95%-confidence intervals (CI) for categorical variables and median and interquartile range (IQR) for continuous variables, are reported. Valid percentages are given, and the frequency of missing data entries per variable is reported. Intergroup comparisons with hypothesis tests were not performed due to limited explanatory power [21].

Associations between hospital admission and in-hospital mortality were assessed via multivariate logistic regression. The included variables were selected based on previous findings in the literature. The resulting crude and adjusted odds ratios are displayed with the 95%-CI. Additionally, a generalized linear mixed effect model with country as random effect was applied. All given *p*-values are not confirmatory but exploratory.

Statistical analyses were performed with SPSS version 27.0 (IBM Inc., Chicago, USA).

## Results

There were 2,794 patients in the data set presenting to an ED with one specified chief complaint. The distribution between men and women was even. Mean age was 77.4 years (SD ± 8.2). Almost every second patient was admitted to hospital and half of the presentations to the ED were out of hours, i.e. after 4 pm. The frequency of risk factors for adverse outcomes in a geriatric population was as follows: *n* = 672 (26.3%) for polypharmacy; *n* = 287 (11.2%) for previous falls; *n* = 627 (24.3%) for the need for home help service; and *n* = 227 (8.6%) for the presence of dementia or Alzheimer’s disease. Table 1 displays patients’ characteristics and chief complaints on ED admission and the clinical course of all patients and hospitalized patients. The five most frequent pre-specified chief complaints were shortness of breath, abdominal pain, chest pain, weakness and fatigue, and extremity pain and numbness. These symptoms represented 59.0% (*n* = 1,648) of the study population, and 81.6% (*n* = 2,280) exhibited the ten most frequent complaints. Supplementary Fig. 1 shows the patterns of the top 5 chief complaints in sex and age subgroups. In very old patients, mental status change and coma replaced chest pain among the top five chief complaints. Leading symptoms can indicate diseases affecting various organ systems. We analyzed emergency department diagnoses according to ICD-10 chapters to determine how heterogeneous the distribution of different diagnostic chapters was in relation to the leading symptoms of the study population. While patients with the chief complaints abdominal pain and chest pain received a diagnosis whose chapter of the ICD-10 catalogue corresponded to the symptoms at ED admission and included roughly two thirds of cases, the diagnoses of patients with the chief complaint extremity pain and numbness as well as weakness and fatigue exhibited a scattered distribution of diagnosis chapters (Table 2).

**Table 1:** Characteristics of the total study population and of hospitalized patients

**Variables**	**Total population** ***n* = 2,794**		**Hospitalized Patients** ***n* = 1,374**	
Age in years (SD)	77.4 (± 8.2)		78.5 (± 8.3)	
	**n (%)**	**95%-CI**	**n (%)**	**95%-CI**
Gender, female	1,401 (50.1) (missing 1)	[48.3–52.0]	639 (46.5) (missing 1)	[43.8–49.2]
Age groups				
65–74y	1,149 (41.1)	[39.3–43.0]	488 (35.5)	[33.0–38.1]
75–84y	1,033 (37.0)	[35.2–38.8]	532 (38.7)	[36.1–41.4]
≥ 85y	612 (21.9)	[20.4–23.5]	354 (25.8)	[23.5–28.2]
Day of Admission				
Monday – Friday	2,133 (76.4)	[74.7–77.9]	1,052 (76.6)	[74.2–78.8]
Weekend	660 (23.6) (missing 1)	[22.1–25.2]	322 (23.4)	[21.2–25.8]
Time of Admission				
08:01–16:00	1,433 (51.3)	[49.4–53.2]	683 (49.7)	[47.0–52.4]
Out of hours*	1,359 (48.7) (missing 2)	[46.8–50.1]	691 (50.3)	[47.6–53.0]
Presenting symptom				
Abdominal pain	368 (13.2)	[11.9–14.5]	145 (10.6)	[9.0–12.3]
Agitation and psychosis	60 (2.1)	[1.6–2.8]	31 (2.3)	[1.5–3.2]
Alcoholic patient	9 (0.3)	[0.2–0.6]	2 (0.2)	[0.0–0.5]
Back pain	69 (2.5)	[1.9—3.1]	17 (1.2)	[0.7–2.0]
Bleeding	138 (4.9)	[4.2–5.8]	52 (3.8)	[2.8–4.9]
Chest pain	271 (9.7)	[8.6–10.9]	113 (8.2)	[6.8–9.8]
Dizziness/Vertigo	115 (4.1)	[3.4–4.9]	32 (2.3)	[1.6–3.3]
Extremity pain and numbness	213 (7.6)	[6.7–8.7]	82 (6.0)	[4.8–7.4]
Fever/Elevated temperature	123 (4.4)	[3.7–5.2]	75 (5.5)	[4.3–6.8]
Headache	78 (2.8)	[2.2–3.5]	23 (1.7)	[1.1–2.5]
Hypotension	8 (0.3)	[0.1–0.6]	4 (0.3)	[0.1–0.7]
Jaundice	7 (0.3)	[0.1–0.5]	6 (0.4)	[0.2–1.0]
Mental status change and coma	130 (4.7)	[3.9–5.5]	94 (6.9)	[5.6–8.3]
Palpitations and Tachycardia	89 (3.2)	[2.6–3.9]	30 (2.2)	[1.5–3.1]
Rash	30 (1.1)	[0.7–1.5]	5 (0.4)	[0.1–0.9]
Seizure	29 (1.0)	[0.7–1.5]	14 (1.0)	[0.6–1.7]
Shortness of Breath	566 (20.3)	[18.8–21.8]	398 (29.0)	[26.6–31.4]
Syncope and near Syncope	126 (4.5)	[3.8–5.4]	56 (4.1)	[3.1–5.3]
Toxic ingestion	17 (0.6)	0.4–1.0]	6 (0.4)	[0.2–1.0]
Change in vision	30 (1.1)	[0.7–1.5]	6 (0.4)	[0.2–1.0]
Weakness and Fatigue	230 (8.2)	[7.2–9.3]	134 (9.8)	[8.2–11.4]
Abnormal findings on examination	88 (3.1)	[2.5–3.9]	49 (3.6)	[2.7–4.7]
Geriatric risk factors				
Home help services utilization	627 (24.3) (missing 211)	[22.6–26.0]	382 (29.4) (missing 73)	[26.9–31.9]
Previous falls	287 (11.2) (missing 223)	[10.0–12.4]	180 (13.9) (missing 79)	[12.1–15.9]
Polypharmacy^~^	672 (26.3) (missing 242)	[24.4–27.9]	379 (29.9) (missing 108)	[27.4–32.5]
Dementia/AD	227 (8.6) (missing 142)	[7.5–9.7]	137 (10.4) (missing 60)	[8.8–12.2]

**Variables**	**Total population** ***n* = 2,794**		**Hospitalized Patients** ***n* = 1,374**	
	**n (%)**	**95%-CI**	**n (%)**	**95%-CI**
Risk profiles				
Geriatric Risk	1,078 (38.6)	[36.8–40.4]	598 (43.5)	[40.9–46.2]
Immunocompromised	1,176 (42.1)	[40.3–44.0]	628 (45.7)	[43.1–48.4]
Cardiovascular Risk	2,101 (75.2)	[73.6–76.8]	1,046 (76.1)	[73.8–78.4]
Hospital stay				
Hospitalized patient	1,374 (49.9) (missing 43)	[48.1–51.8]	1,371 (100)	
Admission to ICU	317 (12.3) (missing 218)	[11.1–13.6]	317 (24.8) (missing 97)	[22.5–27.3]
In-Hospital-Mortality	246 (9.7) (missing 256)	[8.6–10.9]	207 (15.6) (missing 44)	[13.7–17.6]
Duration of stay				
LOS-ED in hours [IQR]	5 [3;9]		6 [3;10]	
LOS-hospital in days [IQR]	5 [1;10] (missing 1,001)		7 [4;13] (missing 121)	

*AD* Alzheimer’s disease, *ED* Emergency Department, *ICU* intensive care unit, *LOS* length of stay, *IQR* Interquartile Range, SD Standard Deviation, *CI* Confidence Interval

^*^Out of hours: 16:01–7:59; ^~^Polypharmacy was defined as the intake of ≥ 5 medications

**Table 2:** Most frequent ICD-10 diagnosis chapters for the five most frequent chief complaints for the complete study sample

**Presenting symptoms**	**Treatment diagnosis by ICD-Chapter (n;%)**
Abdominal pain (*n* = 368, missing 1)	1. Digestive (256; 69.8) 2. Genitourinary (66; 18.0) 3. Circulatory (13; 3.5)
Chest pain (*n* = 271)	1. Circulatory (162; 59.8) 2. Musculoskeletal (49; 18.1) 3. Respiratory (21; 7.7)
Extremity pain and numbness (*n* = 213, missing 1)	1. Musculoskeletal (53; 25.0) 2. Circulatory (38; 17.9) 3. Nervous (35; 16.5) 4. Skin (33; 15.6)
Shortness of breath (*n* = 566, missing 2)	1. Infectious (200; 35.5) 2. Respiratory (187; 33.2) 3. Circulatory (129; 22.9)
Weakness and fatigue (*n* = 230; missing 2)	1. Infectious (69; 30.3) 2. Genitourinary (29; 12.7) 3. Nervous (26; 11.4)

*ED* Emergency Department, *ICD-10* International Classification of Diseases Version 10 Digestive ICD-10 Chapters K00–K93; Genitourinary ICD-10-Chapters N00-N99; Circulatory I00-I99; Musculoskeletal M00-M99; Respiratory J00-J99; Nervous G00-G99; Skin L00-L99

While the chief complaints abdominal pain and chest pain exhibit a clear leading diagnosis chapter that corresponds to the symptoms at ED admission and includes roughly two thirds of cases, the diagnoses assigned to patients with the chief complaints extremity pain and numbness as well as weakness and fatigue have a scattered distribution of diagnosis chapters. This observation suggest that these chief complaints subsume distinct medical conditions and require careful processing in the ED.

**Fig. 2:**
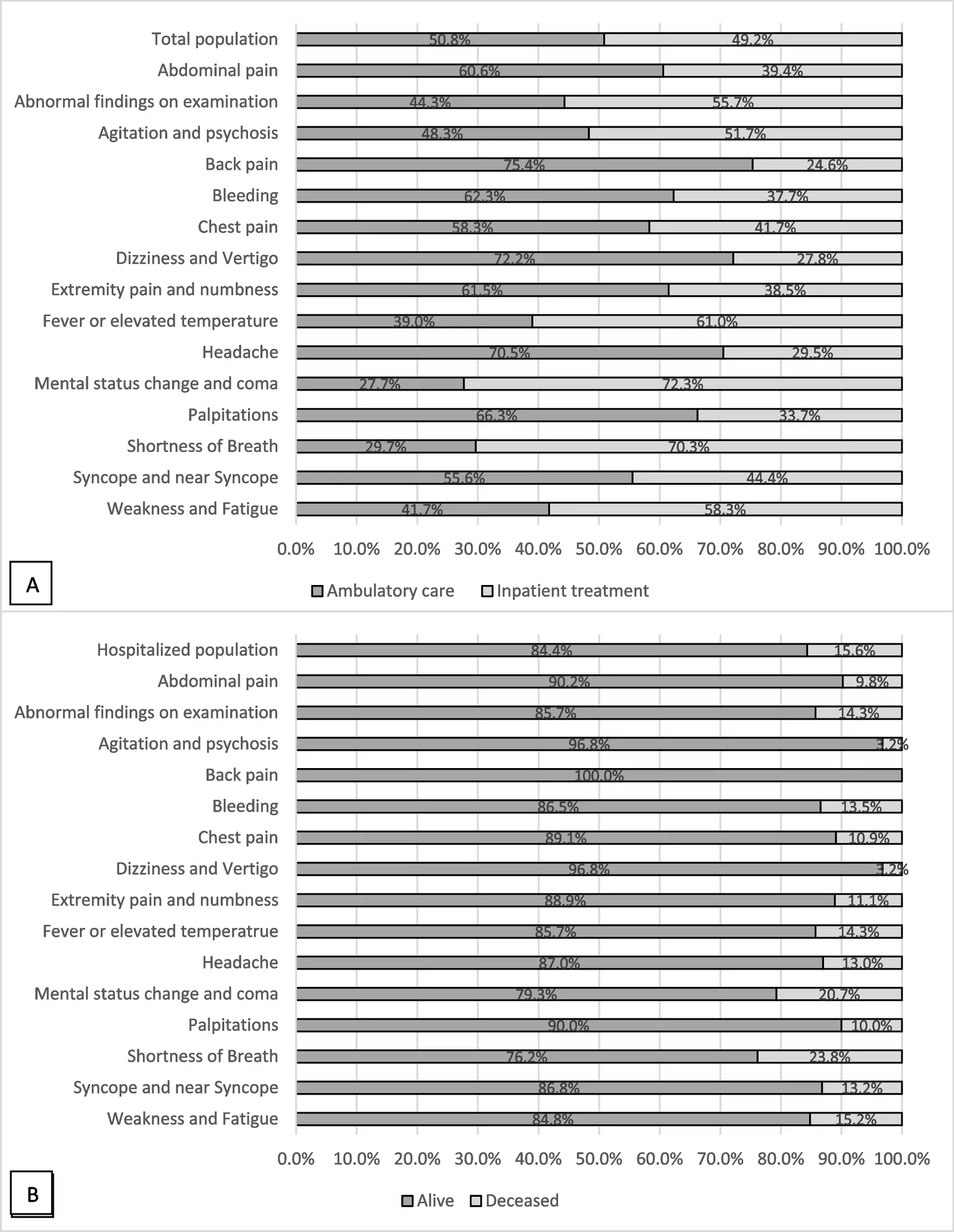
Admission to hospital and in-hospital mortality by chief complaints **A**: Share of admitted patients by chief complaint **B**: Share of deceased inpatients by chief complaint of hospitalized patients. Only chief complaints with *n* > 30 cases were included in the figure. The respective results for not illustrated complaints: complaint (admission rate/mortality rate): alcoholic patient *n* = 9 (39.4%/9.8%); Hypotension *n* = 8 (50.0%/0.0%); Jaundice *n* = 7 (85.7%/50%); Rash *n* = 30 (16.7%/-); Seizure *n* = 29 (48.3%/7.1%); Toxic ingestion *n* = 17(35.3%/16.7%); Change in vision *n* = 30 (20.0%/-)

In patients with a single chief complaint as free text variable, neurologic or stroke-like symptoms, genitourinary symptoms, and flu-like or COVID-19-associated symptoms were the most frequent entries (data not shown). Figure 2 illustrates the different proportions/shares of hospital admission and in hospital mortality with regard to all studied chief complaints with more than 30 entries. Admission rates of the given chief complaints varied between 20.0% – 72.3% and in-hospital mortality was highest in patients presenting with shortness of breath (23.8%) mental status change and coma (20.7%), and weakness and fatigue (15.6%), while no patients admitted for back pain died (Fig. 2).

Binary logistic regression analyses for factors associated with hospital admission in the complete cohort revealed that polypharmacy, multimorbidity, home help service utilization, previous falls, and dementia as well as highest age (i.e. 85 years and older) increased the probability of being admitted (Table 3). This effect was stable in the adjusted regression analyses (OR 1.6. [1.3–1.9]; OR 1.4 [1.2–1.8] respectively) and in the mixed effect model with country as random effect (OR 1.5 [1.2–1.8]; 1.4 [1.2–1.8] respectively) (Table 3) and persisted for both any admission and admissions to normal ward (data not shown). Women showed consistently lower odds for hospitalization (OR 0.7 [0.6–0.9]) (Table 3). The analyses for in-hospital mortality in hospitalized patients showed that the chief complaint shortness of breath increased the mortality in all admissions (OR 2.2 [1.5–3.2]) (Table 4).

**Table 3:** Analysis of parameters associated with admission to hospital with binary logistic regression and generalized linear mixed model (GLMM)

**Any admission**	**Admission to hospital crude**			**Admission to hospital adjusted**			**Admission to hospital GLMM**		
	**Crude OR**	**95%-CI**	***p*-value**	**AOR**	**95%-CI**	***p*-value**	**AOR**	**95%-CI**	***p*-value**
Age ≥ 85	1.6	1.4–1.9	< 0.001	1.4	1.2–1.8	< 0.001	1.4	1.2–1.8	< 0.001
Gender, female	0.8	0.7–0.9	< 0.001	0.7	0.6–0.8	< 0.001	0.7	0.6–0.9	< 0.001
Geriatric Risk, yes	1.6	1.4–1.8	< 0.001	1.6	1.3–1.9	< 0.001	1.5	1.2–1.8	< 0.001
Polypharmacy, yes	1.5	1.2–1.7	< 0.001	1.3	1.1–1.6	0.007	1.2	1.0–1.5	0.061
Admission at weekend, yes	1.0	0.8–1.2	0.839	0.9	0.8–1.1	0.426	0.9	0.8–1.1	0.477
Admission out-of-hours, yes	1.1	1.0–1.3	0.123	1.1	0.9–1.3	0.212	1.2	1.0–1.4	0.075
Abdominal pain	0.6	0.5–0.8	< 0.001	0.9	0.7–1.1	0.227	0.9	0.7–1.1	0.236
Chest pain	0.7	0.5–0.9	0.004	0.9	0.7–1.2	0.478	0.9	0.7–1.2	0.410
Extremity pain and numbness	0.6	0.5–0.8	0.001	0.8	0.6–1.1	0.097	0.8	0.5–1.0	0.090
Shortness of breath	3.2	2.6–3.9	< 0.001	3.0	2.4–3.8	< 0.001	3.3	2.6–4.2	< 0.001
Weakness and fatigue	1.6	1.2–2.1	0.001	1.9	1.4–2.6	< 0.001	2.0	1.5–2.8	< 0.001

*OR* Odds ratio, *CI* Confidence interval, *AOR* adjusted odds ratio

**Table 4:** Analysis of parameters associated with in-hospital mortality with binary logistic regression and generalized linear mixed model (GLMM)

**All admissions (normal ward and ICU)**	**In-hospital mortality crude**			**In-hospital mortality adjusted**			**In-hospital mortality GLMM**		
	**Crude OR**	**95%-CI**	***p*-value**	**AOR**	**95%-CI**	***p*-value**	**AOR**	**95%-CI**	***p*-value**
Age ≥ 85	1.4	1.0–1.9	0.055	1.4	1.0–2.0	0.086	1.6	1.1–2.3	0.019
Gender, female	0.9	0.6–1.2	0.380	0.8	0.6–1.1	0.103	0.7	0.5–1.0	0.030
Geriatric Risk, yes	1.2	0.9–1.6	0.287	1.2	0.8–1.6	0.362	1.4	1.0–2.0	0.082
Polypharmacy, yes	0.6	0.5–0.9	0.018	0.6	0.4–0.9	0.010	0.7	0.5–1.1	0.116
Admission at weekend, yes	1.1	0.8–1.6	0.485	1.0	0.7–1.4	0.985	1.0	0.7–1.4	0.933
Admission out-of-hours, yes	1.5	1.0–1.8	0.043	1.3	1.0–1.8	0.087	1.2	0.9–1.7	0.211
Abdominal pain	0.6	0.3–1.0	0.046	0.8	0.4–1.4	0.405	0.8	0.4–1.4	0.386
Chest pain	0.6	0.3–1.2	0.163	0.9	0.5–1.8	0.808	0.8	0.4–1.7	0.605
Extremity pain and numbness	0.7	0.3–1.3	0.257	0.8	0.4–1.9	0.675	0.7	0.3–1.6	0.419
Shortness of breath	2.3	1.7–3.1	< 0.001	2.2	1.5–3.2	< 0.001	1.8	1.2–2.7	0.002
Weakness and fatigue	1.0	0.6–1.6	0.906	1.1	0.6–2.1	0.671	1.0	0.5–1.8	0.914

*OR* Odds ratio, *AOR* adjusted odds ratio, *CI* Confidence interval

## Discussion

This secondary analysis of the EGERS cohort, focusing on older emergency department (ED) patients across multiple European countries, identified shortness of breath, abdominal pain, chest pain, weakness and fatigue, and extremity pain and numbness as the five most frequent chief complaints. Shortness of breath was the most common presenting symptom and was significantly associated with increased in-hospital mortality, highlighting the critical nature of this complaint among older adults. Highest age (≥ 85), the presence of geriatric risks, polypharmacy, and shortness of breath increased the odds of being admitted to hospital, while female sex was negatively associated with admission.

An important contextual factor for interpreting our findings is that data collection (October 19–November 30, 2020) coincided with the rapid escalation and peak of the COVID-19 wave in Europe, a period during which several participating countries implemented national or partial lockdowns and the population remained largely unvaccinated. Thus, resulting in high in hospital-mortality rates, increased urgency of presentation and reduced presentations of non-COVID-19-related medical conditions in EDs [18, 19, 22, 23]. It is therefore plausible that the observed distribution of chief complaints reflects pandemic-era case mix and health-system strain rather than the usual epidemiology of non-traumatic chief complaints among older ED patients because the pandemic likely altered the underlying population presenting to the ED rather than simply shifting outcomes within a stable population,

However, the prominence of shortness of breath as a leading complaint in older patients aligns with pre-pandemic studies and has been reported as a predictor of one-year mortality [6, 24]. The high number of ICU admissions and the high in-hospital mortality on regular wards and in patients presenting with shortness of breath are likely reflections of pandemic-related outcomes.

Older patients present with less pronounced or atypical symptoms to ED despite severe underlying medical conditions and established tools for risk stratification fail to detect severely ill patients [3, 25]. In the EGERS cohort the chief complaint weakness and fatigue represented the so-called non-specific complaints (NSC). In accordance to previous studies both admission rate and in-hospital mortality in the group of patients that presented with weakness and fatigue are among the highest observed in all chief complaints [4, 6, 15]. Despite the fact that there is no uniform definition of NSC and different authors either use weakness or a set of certain diagnoses and symptoms [14, 25], they are associated with adverse outcomes [26]. Our data underlines the conclusion of Kemp et al. that “NSC should be considered a major emergency presentation” [15]. This underscores the need for increased clinician awareness and targeted training to recognize weakness and fatigue as a potentially time critical and high-risk presentation in older patients in ED, warranting a lower threshold for comprehensive work-up and senior review.

In line with the growing body of evidence on aging populations, our analysis demonstrates that risk factors – such as polypharmacy, prior falls, dementia, and the need for home help services – substantially increase the likelihood of hospital admission [8, 11, 27].

The definition of geriatric risks that was applied in this analysis included previous falls, the presence of a diagnosis of dementia or Alzheimer’s disease or the utilization of home help service. The screening for cognitive impairment, care dependency and reduced mobility as well as the assessment of frailty on ED admission proofed to predict health outcomes [28, 29]. Future studies incorporating comprehensive frailty assessment tools could provide further clarity on how frailty and comorbidities drive admission decisions in older ED patients. ED care for older adults need to include a comprehensive assessment of underlying conditions that impact health outcomes.

Hospital admissions in older patients are associated with health risks such as delirium, nosocomial infections and falls [12]. In this cohort almost half of the population were admitted, It is not clear whether all reported admissions were for purely medical reasons or whether psychosocial reasons such as inadequate nursing care were also drivers for admission. Additionally, potential care provision gaps in the ambulatory sector that strain ED and hospital services may contribute to ED presentations of older adults, especially those with reduced mobility like described in the study of Sengüldür and al. on bedridden patients [30]. While there is evidence for the safety of alternatives to hospital admissions as ‘hospital at home’ or ‘virtual wards’ they are not yet integrated in all European health care systems [31].

Gender affects the urgency and the outcomes of ED treatments. In this study women were consistently less likely to be admitted compared to men. This is in line with former studies that reported higher urgency levels as measured by triage categories, higher admission rates and higher mortality rates in male patients [32, 33].

### Strengths and limitations

This multicenter study is the first to report chief complaints of older ED patients throughout Europe and links them to clinical outcome measures. The study sample reflects a complete capture of the geriatric population in the time frame of the study in the participating EDs. However, the nature of observational studies and the limited period of recruitment hampers the generalizability of the findings. The most pronounced influence is that data collection occurred during the peak of the second European COVID-19 wave (October 19–November 30, 2020), a period characterized by lockdowns, altered care-seeking behavior, shifted triage and admission thresholds, constrained hospital/ICU capacity, and a largely unvaccinated population. These pandemic-related factors likely influenced both the distribution of chief complaints and associated outcomes, including in-hospital mortality. As the report form did not include a standardized assessment of chronic conditions as e.g. the Charlson comorbidity index the comparability between the populations of the various countries is limited to demographic, ED and clinical variables. The chief complaints in the data set left out several relevant categories such as urological symptoms and neurological or stroke like symptoms of which the latter are associated with poorer outcomes with regard to mortality and admission rates. Patients with these symptoms are not represented in this study population. Mortality data was restricted to in-hospital mortality, this likely underestimates short-term mortality after ED discharge and may limit comparability across countries and hospitals with differing admission and discharge practices. Thus, not reflecting post-discharge events.Moreover, the underreporting of certain geriatric risk factors, such as polypharmacy, due to variability in documentation practices across study centers, may have resulted in an incomplete understanding of the full scope of risks among the study population.

## Conclusion

In conclusion, older adults presenting to European EDs frequently require hospital admissions, particularly very old patients. Chief complaints may not point to the underlying condition or reflect its severity underscoring the need for targeted training of ED staff to recognize high-risk presentations and screen for risk factors for adverse health outcomes. In particular, older adults presenting with shortness of breath, non-specific complaints, and geriatric risk indicators require early recognition through structured evaluation. Future studies should evaluate geriatric-specific pathways for older adults in ED and explore alternative care models to hospital admissions that improve healthcare for older patients and reduce admissions, while maintaining high-quality of care.

## Supplementary Information

Supplementary Material 1: Supplementary Fig. 1: 5 most frequent chief complaints and their relative frequency divided by sex and age groups.

Supplementary Material 2: Supplementary Table 1: Epidemiology, clinical course and outcome variables of the total study population stratified by the five most frequent chief complaints. *for patients admitted to hospital only, ^#^one missing data entry, IQR Interquartile Range; ED Emergency Department; LOS Length of Stay; ICU Intensive Care Unit.

## Data Availability

The datasets generated and/or analysed during the current study are not publicly available due to data protection legislaton but are available from the author Zerrin D. Dündar on reasonable request.

## Abbreviations

CI: Confidence Interval
ED: Emergency Department
EGERS: Epidemiology of Geriatric Patients Presenting to Emergency Departments in Europe
IQR: Interquartile range
LOS: Length of stay
NSC: Non-specific complaints
OR: Odds ratio
SD: Standard deviation
