# Supplementary material for: Non-traumatic chief complaints in older patients in European emergency departments and associated outcomes during the Covid-19 pandemic – a secondary analysis of the cross sectional EGERS study
Source: BMC Geriatr. 2026 Sep 5;26:1138. doi: 10.1186/s12877-026-08158-3 (PMC13546510; doi:10.1186/s12877-026-08158-3)
Supplement: Supplementary file 1 [file 12877_2026_8158_MOESM1_ESM.docx]

Supplementary Figure 1:

Supplementary Figure 1: 5 most frequent chief complaints and their relative frequency divided by sex and age groups.
