# Supplementary material for: Non-traumatic chief complaints in older patients in European emergency departments and associated outcomes during the Covid-19 pandemic – a secondary analysis of the cross sectional EGERS study
Source: BMC Geriatr. 2026 Sep 5;26:1138. doi: 10.1186/s12877-026-08158-3 (PMC13546510; doi:10.1186/s12877-026-08158-3)
Supplement: Supplementary file 2 [file 12877_2026_8158_MOESM2_ESM.docx]

**Supplementary Table 1** Epidemiology, clinical course and outcome variables of the total study population stratified by the five most frequent chief complaints

|  | Shortness of Breath  n=566 | Abdominal pain  n=368 | Chest pain  n=271 | Weakness and Fatigue  n=230 | Extremity pain and numbness  n=213 | Total study population  n=2,794 |
| --- | --- | --- | --- | --- | --- | --- |
| Age, median [IQR] | 78 [72;85] | 75 [69;82] | 74 [69;80] | 78 [72;86] | 76 [69;83] | 77[71;84] |
| Age groups n (%)  65 – 74  75 – 84  ≥85 | 203 (35.9)  221 (39.0)  142 (25.1) | 180 (48.9)  117 (31.8)  71 (19.3) | 139 (51.3)  95 (35.1)  37 (13.7) | 90 (39.1)  73 (31.7)  67 (29.1) | 98 (46.0)  71 (33.3)  44 (20.7) | 1,149 (41.1)  1,033 (37.0)  612 (21.9) |
| Gender, women n (%) | 266 (47.1) ^#^ | 191 (51.9) | 126 (46.5) | 102 (44.3) | 121 (56.8) | 1,401 (50.1) |
| LOS ED in hours median [IQR] | 6 [4;10] | 5 [3;8] | 5 [3;8] | 7 [4;12] | 4 [3;8] | 5 [3;9] |
| Polypharmacy n (%) | 165 (31.2)  missing 37 | 74 (20.6)  missing 9 | 90 (33.8)  missing 5 | 52 (25.0)  missing 22 | 59 (29.5)  missing 13 | 672 (26.3)  missing 242 |
| Median number of medications median [IQR] | 3 [2;5]  missing 37 | 2 [1;4]  missing 9 | 3 [1;5]  missing 5 | 3 [1;5]  missing 22 | 3 [1;5]  missing 13 | 3 [1;5]  missing 242 |
| Dementia n (%) | 53 (9.5)  missing 11 | 20 (5.5)  missing 2 | 9 (3.3) | 28 (12.4)  missing 6 | 16 (7.8)  missing 9 | 227 (8.6)  missing 142 |
| Falls n (%) | 53 (9.7)  missing 19 | 23 (6.6)  missing 18 | 13 (4.9)  missing 7 | 28 (12.2)  missing 4 | 33 (16.8)  missing 17 | 287 (11.2)  missing 223 |
| Home help service n (%) | 182 (33.0)  missing 15 | 65 (18.3)  missing 12 | 34 (13.1)  missing 11 | 69 (30.5)  missing 4 | 38 (19.3)  missing 16 | 627 (24.3) missing 211 |
| Hospitalized patients n (%) | 398 (71.8)  missing 12 | 145 (39.8)  missing 4 | 112 (41.3)  missing 1 | 134 (58.3)  missing 9 | 82 (38.5  missing 1 | 1,374 (49.9)  missing 43 |
| ICU admission* n (%) | 144 (26.4)  missing 21 | 15 (4.1) | 43 (15.9)  missing 8 | 23 (10.0)  missing 11 | 11 (5.4)  missing 11 | 317 (12.3)  missing 218 |
| LOS hospital in days median [IQR]* | 10 [6;16]  missing 20 | 3 [1;8] | 2 [1;5] | 7 [2;12] | 3 [1;9] | 5 [1;10]  missing 1,001 |
| In-hospital mortality* n (%) | 92 (23.8)  missing 12 | 18 (5.4)  missing 34 | 12 (4.4)  missing 32 | 28 (14.2)  missing 33 | 10 (5.0)  missing 14 | 246 (9.7)  missing 256 |

*****for patients admitted to hospital only, ^#^one missing data entry, IQR Interquartile Range; ED Emergency Department; LOS Length of Stay; ICU Intensive Care Unit
